# Predictors of Participation in the U.S. Department of Agriculture Summer Meal Programs: An Examination of Outreach Strategies and Meal Distribution Methods During COVID-19

**DOI:** 10.1016/j.focus.2023.100124

**Published:** 2023-06-21

**Authors:** Brooke L. Bennett, Juliana F.W. Cohen, Tatiana Andreyeva, Julia Esposito, Kara Burkholder, Sandra M. Chafouleas, Marlene B. Schwartz

**Affiliations:** 1 Rudd Center for Food Policy and Health, University of Connecticut, Hartford, Connecticut; 2 Center for Health Inclusion, Research and Practice (CHIRP), Merrimack College, North Andover, Massachusetts; 3 Department of Public Health and Nutrition, Merrimack College, North Andover, Massachusetts; 4 Department of Nutrition, Harvard T.H. Chan School of Public Health, Boston, Massachusetts; 5 Department of Agricultural and Resource Economics, Rudd Center for Food Policy and Health, University of Connecticut, Hartford, Connecticut; 6 Department of Educational Psychology, University of Connecticut, Storrs, Connecticut; 7 Department of Human Development and Family Sciences, University of Connecticut, Storrs, Connecticut

**Keywords:** Summer meal programs, U.S. Department of Agriculture, National School Lunch Program, food insecurity, school nutrition, outreach strategies

## Abstract

**Introduction:**

Significantly fewer children participate in the U.S. Department of Agriculture–sponsored summer meal programs than in the federal school meal programs during the academic year. During the summer of 2021, several pandemic-related waivers supported more flexible operations for summer meal programs, such as allowing grab-and-go meals and the distribution of meals for multiple days at once. This study assessed how summer meal site characteristics and geographically targeted outreach methods were associated with summer meals served in 2021 in Connecticut.

**Methods:**

Weekly meal count data were requested from all sponsors of the Summer Food Service Program and the National School Lunch Program Seamless Summer Option. Data were received from 78 sponsors with 763 sites. Geographically targeted outreach (e.g., billboards, bus ads, and flyers) was tracked by location each week. Mixed methods ANOVA was used to examine the predictive value of outreach efforts, program characteristics, and meal distribution methods on meals served each week. The program characteristics examined included the hours open per week, the number of weeks serving meals, the maximum number of meals distributed at one time, and the number of open and closed sites in a school district.

**Results:**

Between June 21 and August 20, 2021, a total of 1,188,669 breakfasts and 1,389,347 lunches were served. Sites that were open more hours per week (mean=7.60 hours per week; range=0.17–35) and gave multiple meals to parents at each visit (mean=4.68 meals; range=1–15) reported greater rates of meal distribution. The outreach efforts assessed were not significantly associated with changes in meal distribution at nearby sites.

**Conclusions:**

The U.S. Department of Agriculture's waivers in response to COVID-19 permitted extended operating hours and multiple-meal distribution. These operational flexibilities should be considered for permanent regulation changes owing to their positive association with summer meal participation.

## HIGHLIGHTS

• Pandemic-related waivers allowed more flexible operations for summer meal programs.
• Sites that were open more hours per week had greater rates of meal distribution.
• Sites that gave parents multiple meals at each visit higher average participation.
• Targeted outreach efforts were not associated with increased participation.
• Several operational waivers should be considered for permanent regulation change.

## INTRODUCTION

Approximately 1 in 6 children in America experienced food insecurity in 2021.^1^ To increase access to nutritious meals during the academic year, the U.S. Department of Agriculture (USDA) provides funds to feed children through the School Breakfast Program and the National School Lunch Program (NSLP). Studies have shown that children who receive free and reduced-price meals from these programs have a lower risk of food insecurity.^2^ During the summer, however, these meals are unavailable and the rates of child food insufficiency increase.^3^^,^^4^

To address the gap in school meal provisions during the summer months, the USDA has 2 federal meal programs: the Summer Food Service Program (SFSP) and the NSLP Seamless Summer Option (SSO). SFSP and SSO program sponsors distribute meals during the summer at pre-registered and approved sites.^5^ The 2 programs have similar regulatory restrictions (e.g., participant eligibility, meal service location restrictions, site eligibility, and meal types), but differ in some administrative procedures (e.g., application process and reimbursement rates). The meal pattern requirements also differ between the 2 programs: SSOs must match the standards of NSLP and School Breakfast Program, whereas SFSPs can choose to meet those standards or follow the slightly more flexible SFSP meal pattern.^6^ Summer meal sites can also be open or closed. Open sites are located in community settings, such as schools, parks, or community centers, and provide meals at no cost to any child aged ≤18 years. Closed sites provide meals for specific students who are enrolled in a program such as a camp or summer school.

Before the coronavirus disease 2019 (COVID-19) pandemic, participation in the summer meal programs was consistently lower than the participation by low-income students in the academic year school meal programs. For example, a 2019 study of summer meal program participation in California found overall uptake was merely 18% of their target population.^7^ A national study in 2019 found that only 13.8 children received lunch in July for every 100 children who received lunch during the school year.^8^ Even the highest-performing states served approximately 1 child over the summer for every 4 who participated in free or reduced-price lunch during the school year (i.e., a 1:4 ratio), whereas the lowest-performing states had ratios closer to 1:10.^8^ Thus, it is important to investigate the ways in which state agencies and summer meal program sponsors can maximize participation in federal meal programs during the summer months.

Several studies have identified a lack of awareness of the summer meal programs as a significant barrier to participation.^9^, ^10^, ^11^ Therefore, 1 potential way to improve participation is through outreach and advertising. A multistate study of strategies used to promote summer meals found that the most common marketing element was paid advertising (e.g., flyers; television, radio, and newspaper ads; signs and billboards), followed by public relations (e.g., events) and direct marketing (e.g., via e-mail, phone, and text).^12^ Indeed, using flyers, billboards, and bus advertisements to advertise summer meals has been a popular strategy in Connecticut and elsewhere,^13^ however, there are financial and time costs for the organizations involved. It is important to know whether these investments are associated with an increase in participation in areas where they are targeted.

Another potential inroad through which to increase participation in summer meal programs is to identify whether different distribution strategies and site-specific characteristics are associated with increased participation. Historically, because of the USDA's regulatory restrictions for the SFSP and SSO, there was little variability in distribution methods to examine; all meals were distributed one at a time to children accompanied by an adult caregiver and were required to be consumed on-site in a congregate setting.

Following the onset of the COVID-19 pandemic in 2020 and the immediate increase in food insecurity,^1^ the USDA released several regulatory waivers in 2020 and 2021 that allowed new flexibility in the summer meal programs. For example, 1 waiver changed area eligibility criteria cut-offs so that districts with free/reduced rates <50% could apply to their state agencies to participate and host meal sites. Additionally, several waivers granted flexibility to sponsors, including permission to serve (1) grab-and-go meals; (2) meals that deviated from the school meal pattern requirements; (3) multiple meals at one time; and (4) meals that could be picked up without the child being physically present.^14^ The implementation of these waivers in 2021 presents a unique opportunity to examine how distribution methods may impact participation.

The aim of this study was to examine the factors associated with participation in federally funded summer meal programs in Connecticut in 2021. First, we examined the association between the presence of advertising in a geographic area and the subsequent levels of meal distribution. We hypothesized that the presence of advertising near a summer meal site would be associated with higher rates of participation. Second, we examined how participation rates were associated with program characteristics and meal distribution methods (hours open per week; the number of weeks serving meals; the maximum number of meals distributed at one time; and the number of open and closed sites in a district). The authors hypothesized that methods that increased convenience for participants would contribute to higher numbers of meals distributed.

## METHODS

This study was deemed exempt from full review by the University of Connecticut IRB.

### Study Sample

There are significant economic disparities across school districts in Connecticut. As of 2019, there were about 2 dozen districts where <15% of students were eligible for free or reduced-price meals and 11 large urban districts where more than two thirds of the students were eligible.^15^ Over 9 weeks in summer 2021 (June 21, 2021–August 20, 2021), there were 92 summer meal sponsors that set up 812 meal sites. Out of the state's 191 school districts, 75 were summer meal sponsors.^16^ Additional sponsors (*n*=17) included camps, charter and technical school districts, community nonprofits, and local government agencies.

### Measures

After the summer meal programs concluded, the authors e-mailed the key contact for each sponsor (frequently the food service director) and requested daily breakfast and lunch distribution counts. Snacks and supper counts were not requested because these programs were less common. The daily meal numbers were collapsed into weekly meal counts for breakfast and lunch.

Data about the site characteristics (i.e., hours and days open; types of meals distributed; open versus closed status) were collected using publicly available lists of summer meal sites and locations provided by the Connecticut State Department of Education. Two data sets were created. The school district-level data set connected 1 school district to the meal count data from all sites (even those with different sponsors) that operated within that school district's geographic attendance zone. The authors included the number of open sites and the number of closed sites within the geographic boundary of each school district as district variables. Other district-level variables included total student enrollment and the percentage of students eligible for free and reduced-price meals (obtained from a national database^17^). The site-level data set included records for each site and the number of hours the site was open per week; whether the site was open or closed; and the maximum number of meals given out at one time (e.g., a bag with 7 meals in it was distributed at 10:00 am on Tuesdays, or 1 lunch was provided at 11:00 am on Wednesday).

Outreach data were provided by End Hunger Connecticut (EHC), a community organization funded by the Connecticut State Department of Education to promote the summer meal program statewide with state-branded materials. EHC tracked the type, amount, and location of weekly outreach efforts. Outreach by EHC included (1) delivering flyers and posters; (2) running bus ads, billboard ads, an ad at a drive-in movie theater, and public-service announcements at Department of Motor Vehicle locations; (3) promoting summer meals at community events; and (4) providing subgrants to food pantries to hand out flyers to their patrons. EHC also worked with the United Way to document the origin of calls to 211, a service designed to connect callers to essential community services. A weekly score was created to indicate the presence or absence of geographically targeted outreach efforts in each community. This was used to assess the short-term impact of the outreach on meal distribution at specific sites. Outreach methods for which geographical reach could not be pinpointed (e.g., social media) were excluded from analyses.

### Statistical Analysis

**Outreach efforts**. To assess the impact of the geographically focused outreach efforts, the authors examined the relationship between each of the types of outreach efforts and the participation at nearby open sites. The reason the authors restricted these analyses to only open sites is that they are available to the public and their daily numbers have the potential to fluctuate because of promotional efforts. In contrast, the number of children served by closed sites typically remains stable because closed sites are camps or programs that require formal enrollment. To examine the association between site meal distributions and local outreach efforts, the week before implementation was compared with the week after implementation using mixed methods ANOVA (with district as a random effect). This analysis accounted for repeated measures within a site; the number of hours a site was open per week; and the percentage of students eligible for free and reduced-price meals in the corresponding school district.

**District-level participation**. Two linear regressions were used to examine the differences in the average district-level percentage of participation (measured by the average number of meals served daily divided by the total number of students eligible for free or reduced-price meals within that district) in summer meal programs for breakfast and lunch by site characteristics. One model included the total number of sites as a predictor and a separate model included the number of both closed and open sites. Both models adjusted for the percentage of students eligible for free and reduced-price meals and the total student enrollment in each school district.

**Site-level participation.** Two mixed methods ANOVAs were used (with school district as a random effect) to assess the predictors of participation at each site. The first model examined the association between average weekly participation (measured by meals served divided by total operating weeks) at all summer feeding sites with the following: (1) the maximum number of meals served at a time; (2) the total number of weeks serving meals; and (3) whether sites were open or closed. The second model examined the association between average weekly participation only at open sites with (1) outreach efforts; (2) the maximum number of meals served at a time; (3) the total number of weeks serving meals; and (4) the hours open per week. Both analyses were adjusted for the percentage of students eligible for free and reduced-price meals in the district where the site was located. The analyses examining individual sites excluded sites identified as outliers (i.e., serving <25 students per week).

## RESULTS

**Figure 1:**
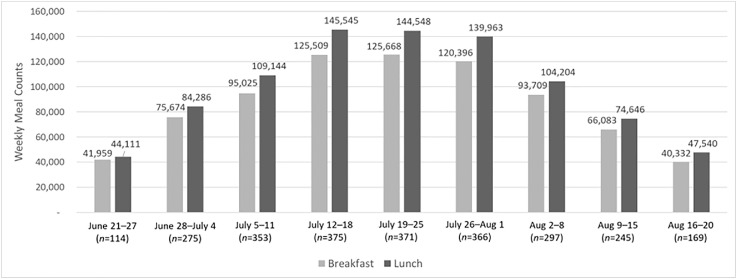
Total weekly summer meals participation among open sites in summer 2021. Aug, August.

**Figure 2:**
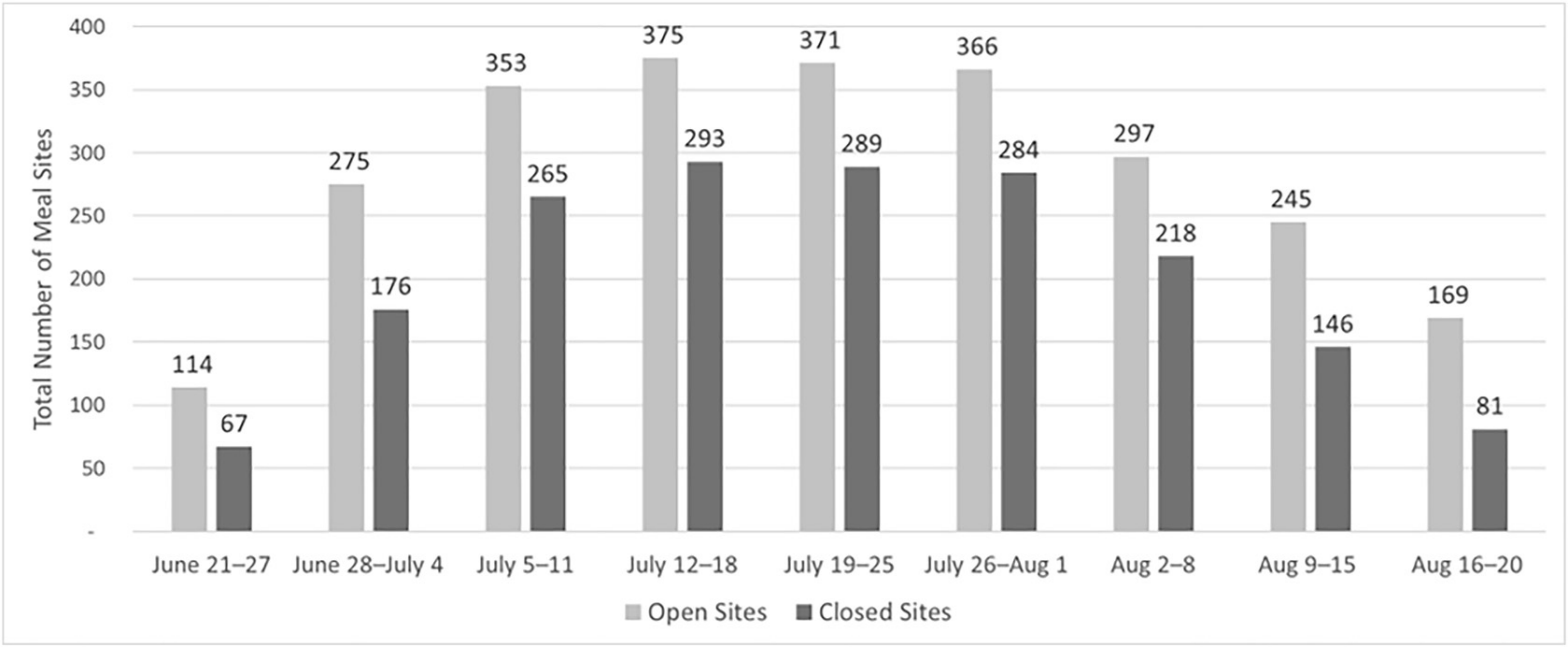
Total number of summer meal sites per week in 2021. Aug, August.

In total, our data represent 2,578,016 meals served in the summer of 2021 in Connecticut, including 1,188,669 breakfasts and 1,389,347 lunches (Figure 1). We received meal count data from 84.8% (*n*=78) of the 2021 program sponsors, representing 93.9% (*n*=763) of the summer meal program sites across 89 districts in Connecticut (Figure 2). The majority (71.4%) of the 14 missing sponsors were non–school district sponsors, such as independent summer camps. Sites were open an average of 7.60 hours per week, with considerable variability (range=0.17–35.00 hours per week). Collectively, the sites utilized the option to distribute multiple meals at once in meal packs; on average, sites distributed 4.68 meals at once (range=1–15 meals).

### Targeted Outreach Efforts

When examining only the open sites, the average number of meals distributed in the weeks following the presence of any of the outreach methods in the community was not significantly different from the meals distributed in prior weeks.

### Predictors of Percent Participation Within School Districts

When examining the factors associated with average district-level percent participation per week, the total number of sites (including both open and closed) was associated with increases in breakfast participation (*β*=0.76; *p*=0.04) and lunch participation (*β*=0.85; *p*=0.02) (Table 1). The total number of weeks a site was open was associated with a higher average participation rate at breakfast (*β*=2.69; *p*=0.01). When examining the effect of site types within the district, the number of closed sites was positively associated with higher participation rates at breakfast (*β*=1.61; *p*=0.01) and lunch (*β*=1.85; *p*=0.005), whereas there was not a statistically significant association between participation rates and the total number of open sites.

**Table 1:** Factors Associated With Average District-Level Percent Participation (*n*=89 Districts)

Number of sites	Breakfast		Lunch	
	β (SE)	*p*-Value	β (SE)	*p*-Value
Total number of sites (open and closed)^a^	**0.76 (0.37)**	**0.04**	**0.85 (0.37)**	**0.02**
Number of closed sites^b^	**1.61 (0.67)**	**0.01**	**1.85 (0.66)**	**0.005**
Number of open sites^b^	0.46 (0.4)	0.30	0.50 (0.41)	0.22
Total # weeks open^a^^,^^b^^,^^c^	**2.69 (1.10)**	**0.01**	1.43 (1.09)	0.20

*Note:* Calculated using linear regression, adjusting for the percentage of students eligible for free and reduced-price meals and total student enrollment within the school district. Boldface indicates statistical significance (*p*<0.05).

a Included in Model 1.

b Included in Model 2.

c Total number of weeks open was included in both models and the outcomes were the same. #, number.

### Predictors of Meals Distributed at Individual Sites

Closed sites served on average 104 fewer breakfasts (*p*<0.001) and 112 fewer lunches (*p*<0.001) per week than open sites (Table 2). Sites that distributed >1 meal per visit served significantly more meals per week than sites that only gave out 1 meal at a time, distributing an average of 10.1 more lunches (*p*=0.01) and 9.5 more breakfasts per week (*p*=0.03).

**Table 2:** Factors Associated With Site-Level Average Weekly Meals Served

Predictors	Breakfast		Lunch	
	β (SE)	*p*-Value	β (SE)	*p*-Value
Closed site (versus open site)	**–103.6 (30.4)**	**0.0006**	**–111.8 (31.9)**	**0.0005**
Maximum # of meals served at one time	**10.1 (4.2)**	**0.01**	**9.5 (4.5)**	**0.03**
Total weeks serving meals	2.9 (7.4)	0.8	1.8 (7.6)	0.8

*Note:* Calculated among sites during the weeks they were open, using mixed model analysis of variance with school district as a random effect, adjusting for the percentage of students eligible for free and reduced-price meals within a school district. Boldface indicates statistical significance (*p*<0.05).

#, number.

When examining only the open sites (Table 3), there was a significant positive association between how many hours they were open per week and the number of meals served at breakfast (*β*=0.13; *p*=0.03) and lunch (*β*=0.14; *p*=0.03). Each additional hour open was associated with serving approximately 8 additional breakfasts and 8 additional lunches per week. Total outreach efforts in the community where the site was located, the maximum number of meals served at one time, and the total number of operational weeks were not significantly related to the distribution rates among the open sites.

**Table 3:** Factors Associated With Site-Level Average Weekly Meals Served Among Open Sites

Predictors	Breakfast		Lunch	
	β (SE)	*p*-Value	β (SE)	*p*-Value
Total outreach	–2.9 (5.6)	0.6	–0.6 (5.8)	0.9
Maximum # of meals served at one time	6.7 (5.3)	0.2	7.3 (5.6)	0.2
Hours open per week^a^	**0.13 (0.06)**	**0.03**	**0.14 (0.06)**	**0.03**
Total weeks serving meals	21.1 (11.8)	0.08	6.0 (7.3)	0.4

*Note:* Boldface indicates statistical significance (*p*<0.05).

a Calculated among sites during the weeks they were open, using mixed model analysis of variance with school district as a random effect, adjusting for the percentage of students eligible for free and reduced-price meals within a school district. #, number.

## DISCUSSION

This study examined the predictors of higher rates of participation in federally funded summer meal programs. The first hypothesis was not supported; the outreach efforts the authors assessed were not predictive of increases in meal distribution rates in nearby meal sites. These findings were unexpected because lack of awareness is regularly cited as a barrier to participation.^9^, ^10^, ^11^ It is noteworthy that the assessed outreach was branded at the state level (i.e., Connecticut Summer Meals), instead of being branded for individual school districts or towns. There is evidence the relationship between parents and their child's school may be key to developing and maintaining connections.^12^^,^^18^^,^^19^ In fact, a recent study found that summer meal program participants reported primarily getting information about these meals directly from their child's school.^20^ The practice of communicating directly with parents could serve sponsors as well, because the USDA requires sponsors to share information about their summer meal programs publicly.^5^ School districts increased their promotion of school nutrition programs on social media during the pandemic,^21^ and a similar strategy may work for the summer meal programs. Future research should examine whether targeted social media campaigns through schools’ websites and social media channels are successful in increasing awareness and participation.

The second aim of this study was to examine the role of different program characteristics and meal distribution methods in predicting summer meal participation rates. The authors found that because of the congregate meal waiver, the average number of meals provided per visit was between 4 and 5; and distributing multiple meals at once was associated with distributing more meals on average each week. Relatedly, closed sites in camps and schools continued to provide congregate meals and distributed significantly fewer meals per week than open sites. The results of this study also demonstrate the importance of the waiver that allowed sites to have extended operating hours. As expected, there was a clear positive relationship between the number of hours a site was open per week and the number of meals distributed.

Taken together, it is possible that increasing the hours of operation and distributing more meals at 1 time were successful strategies because they enhanced the level of convenience for participants. For example, instead of 2 daily trips to get breakfast and lunch, parents could come to a site once or twice a week and still get enough meals for the full week. This is consistent with the previous studies where participants described barriers to the summer meal program that included a lack of transportation^19^^,^^22^^,^^23^ and the limited times of day that the meals were available.^20^ It is important to note, however, that longer hours of operation come with an administrative cost. These findings suggest this cost may be offset by increased participation and therefore increased reimbursement. Future research could conduct a cost-benefit analysis to determine how long a site can remain open before encountering diminishing financial returns.

Several other operational characteristics were associated with meal distribution. First, the number of weeks of operation was associated with a higher rate of breakfast participation. Second, each additional site available in a district corresponded to a 1% increase in total meals distributed. Although a 1% change may seem small, this translates to an additional 25,780 meals served. Third, the number of closed sites within a district was associated with greater meal distribution. This was unexpected because closed meal sites require enrollment in a program such as a summer school or camp. At the same time, meal distribution numbers for these sites tend to remain stable over time, and it is possible that a greater number of closed sites translated to more families in that district being aware of summer meal programs. Future research should examine the mechanism through which closed sites contribute to overall meal distribution.

These findings have implications for federal and state policymakers. In previous research, both participants and sponsors underscored the utility of the USDA's COVID-19 regulatory waivers. Sponsors liked the increased flexibility^23^, ^24^, ^25^ and the ability to innovate to reach more participants.^9^^,^^19^ Families highlighted the importance of being able to pick up multiple meals in a grab-and-go setting as they decided to participate in the program.^20^ The findings of this study support these reports and provide evidence that the changes to program structure permitted by the COVID-19 regulatory waivers are linked to greater overall meal distribution. Therefore, policymakers should consider incorporating these waivers into new regulations.

### Limitations

There were several limitations to this study. First, although the response rate was quite high at 85%, the authors excluded the summer meal programs that did not provide their meal counts, which may have changed the pattern of the results. Next, in this time-bound examination of promotional efforts on meals distributed, the authors used a 1 week lag time to connect each outreach effort with a potential change. However, it is possible that some outreach efforts led to changes 2 or more weeks later. This study was also a natural experiment, so the outreach strategies were not structured to allow comparisons between matched intervention and control communities. A future controlled experiment could systematically implement and measure the short-term and cumulative impact of outreach methods such as bus signs and billboards.

Lastly, this study was conducted during the second summer of the COVID-19 pandemic. Participation rates may have been higher because of a greater need for summer meals resulting from factors related to the pandemic. Alternatively, the waivers may have allowed families struggling with food insecurity to access meals for the first time because of the expanded hours and noncongregate meals. The USDA waivers that expanded district eligibility were also in place, and future research should continue to examine the importance of area eligibility waivers.^26^ Future research should also examine the importance of the geographic placement of summer meal sites^27^^,^^28^ on access and participation.

## CONCLUSIONS

To the best of our knowledge, this study provides the first investigation to examine how outreach efforts, site characteristics, and distribution methods predict participation in the summer meal program. The results demonstrate the need to develop and assess effective outreach and communication strategies to promote the summer meal programs. These findings also underscore the critical role that the USDA's COVID-19 waivers played in feeding children during the summer of 2021. There is evidence to support updating the summer meal regulations to incorporate the elements of the waivers that have been linked to higher participation. These changes will give sponsors more flexibility in how they operate their summer meal program and serve their communities.

## CRediT authorship contribution statement

**Brooke L. Bennett:** Conceptualization, Data curation, Investigation, Methodology, Project administration, Writing – original draft. **Juliana F.W. Cohen:** Conceptualization, Data curation, Formal analysis, Methodology, Visualization, Writing – review & editing. **Tatiana Andreyeva:** Conceptualization, Methodology, Writing – review & editing. **Julia Esposito:** Data curation, Project administration, Writing – original draft. **Kara Burkholder:** Data curation, Project administration. **Sandra M. Chafouleas:** Conceptualization, Funding acquisition. **Marlene B. Schwartz:** Conceptualization, Funding acquisition, Methodology, Supervision, Writing – review & editing.
